# Surgical extraction after thrombosis around the Avalon dual lumen cannula

**DOI:** 10.1308/003588414X13824511649814

**Published:** 2014-01

**Authors:** V Kalem, D Buchwald, J Strauch, A Sidiropoulos, R Meindl, TA Schildhauer, J Swol

**Affiliations:** University Hospital Bergmannsheil, Bochum,Germany

**Keywords:** Extracorporeal membrane oxygenation, Cannula, Thrombosis

## Abstract

The use of a dual lumen cannula (DLC) for venovenous extracorporeal membrane oxygenation (ECMO) has several advantages and reports of complications are rare. We present a case of thrombosis around and inside the Avalon Elite™ bicaval DLC (Avalon Laboratories, Rancho Dominguez, CA, US), for which simple removal by retraction was impossible.

A 30-year-old man had experienced an unstable C6/7 fracture with spinal contusion and haematoma in the spinal canal with incomplete neurological paraplegia and thoracic trauma. He developed acute respiratory failure due to posttraumatic systemic inflammatory response syndrome and venovenous extracorporeal membrane oxygenation (ECMO) support was indicated. The cannulation was performed with an Avalon Elite™ cannula (31Fr) in the right jugular vein under fluoroscopy. After 18 days of ECMO therapy, despite the continuous administration of heparin (400iu/h), ECMO was discontinued because of the formation of a massive thrombus in the oxygenator. At that time, the patient’s haemodynamic and respiratory parameters were stable, and we were able to induce a rapid weaning from ECMO. The surgical removal of the cannula became necessary and was performed using a small neck incision without complications.

We report this case to emphasise that any resistance encountered during an attempt to extract the Avalon Elite™ cannula may cause serious complications. In such cases, surgical removal must be considered.

Major trauma patients may develop acute respiratory distress syndrome (ARDS) along with systemic inflammatory response syndrome (SIRS) or as a result of direct lung tissue injury.^1^ Lung protective ventilation (with a tidal volume of ≤4–6ml/kg/ideal body weight) is recommended for ARDS treatment.^2^ At our level 1 trauma centre, we provide early venovenous extracorporeal membrane oxygenation (ECMO) therapy when lung protective ventilation is no longer possible.^3,4^ Dual lumen cannulas (DLCs) are applied via the internal jugular vein.^5^ Reports of complications from the use of the Avalon Elite™ cannula are rare.^6^ However, a case of a right ventricular rupture during the insertion of this cannula has been reported. This procedure should therefore be performed under visual control (such as fluoroscopy or echocardiography).^6,7^

## Case history

A 30-year-old man (95kg, 188cm) was involved in a serious car accident. He was conscious at the scene but had no sensation in his legs. On admission, he was intubated and sedated, and his cardiorespiratory parameters were stable. Whole body computed tomography (CT) and magnetic resonance imaging revealed an unstable C6/7 fracture and widescale thoracic trauma (Table 1).

**Table 1:** Criteria for venovenous extracorporeal membrane oxygenation treatment in trauma patients in our centre

Parameter	Threshold
Ventilation time in non-lung protective area	>8–12 hours
Fraction of inspired oxygen	≥0.6
Tidal volume	≥4–6ml/kg/ideal body weight
Inspiratory pressure	≥30mmHg
pH	<7.25
Oxygen partial pressure	<60mmHg

After the diagnostic procedures, the patient was transferred to our level 1 trauma centre for surgery but the operation was interrupted when the patient’s respiratory status deteriorated and hypoxemia ensued. The initial chest x-ray showed no signs of an acute pathological condition. The postoperative vital signs and blood gas parameters were satisfactory.

Within the next 40 hours, the patient developed ARDS due to posttraumatic SIRS. Subsequently, severe hypoxemia (pO_2_ <60mmHg), an increased demand for FiO_2_ (from 0.3 to 0.6) and complete atelectasis of a third of the lung tissue in both lungs occurred. As the lung function was impaired, the prone position and venovenous ECMO support were indicated. A 31Fr Avalon Elite™ cannula was applied owing to the size of the patient. This was inserted through the right internal jugular vein into the correct position under fluoroscopy and ultrasonography control.

Initially, the patient’s oxygenation improved significantly under ECMO support. Despite this, over the following days, his condition did not continue to improve despite escalations of the ECMO and respiratory therapies. Reviewed under fluoroscopy, the infusion port was not rotated correctly; the flow port did not point precisely towards the tricuspid valve. The rotation of the cannula was corrected immediately without any complications. After this adjustment, the ECMO and respirator parameters improved.

After 18 days, ECMO was discontinued because of the formation of a massive thrombus in the cannula and the oxygenator. As the patient was already being weaned and exhibited acceptable arterial blood gas parameters, the ECMO circuit was not renewed.

**Figure 1:**
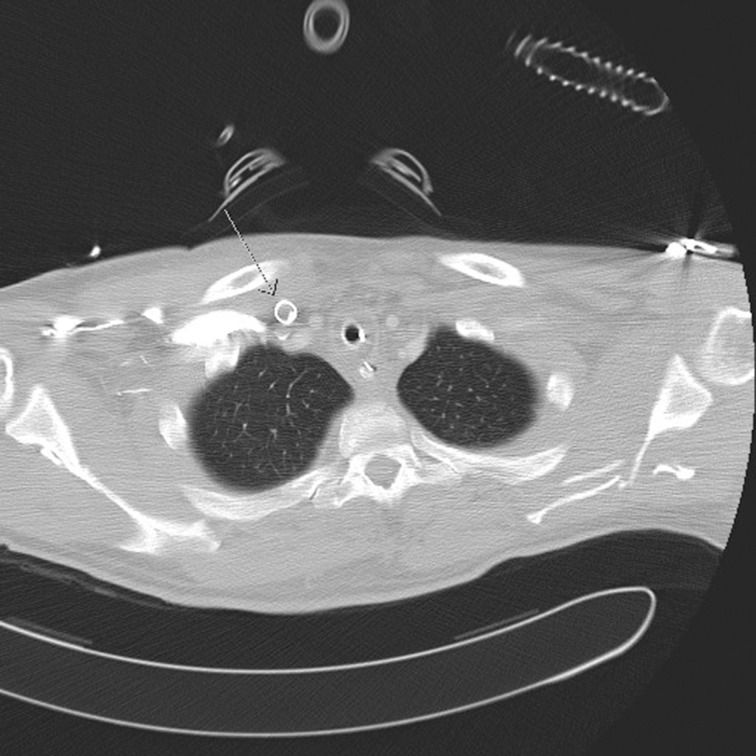
Computed tomography showing the entry of the cannula into the jugular vein with a partial thrombus in the lumen. However, there is no sign of a thrombus or adhesion around the cannula (arrow)

On attempting to extract the Avalon Elite™ cannula from the internal jugular vein, elastic resistance was experienced. CT demonstrated a thrombus in the cannula although the reason for the adhesion of the cannula could not be revealed (Fig 1). Surgical removal of the cannula was therefore inevitable. To date, there have been no cases reported of a thrombosis inside and around an ECMO cannula in the internal jugular vein that required surgical removal of the cannula. It is known that a thrombosis in the atrium after catheterisation can lead to open heart surgery.^8^ However, in this case, the cannula could be removed via a small skin incision beginning slightly caudal to the cannula entry site.

**Figure 2:**
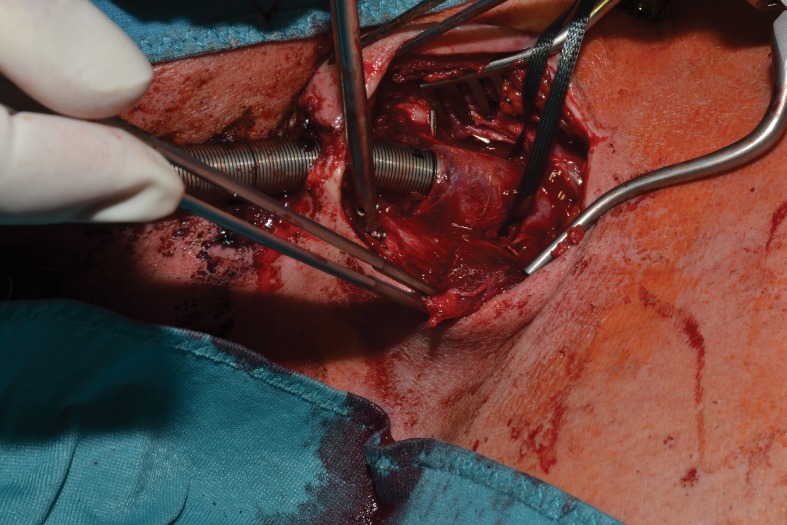
Adherence at the entry point of the cannula into the jugular vein while the cannula is being slightly pulled

Preparation continued until the jugular vein was exposed with the cannula entry point. There was adherence between the vein wall and the cannula due to thrombosis around the cannula (Fig 2). The vessel flow was stopped and after a short vertical incision in the jugular vein, the cannula could be removed with cautious sharp and blunt preparations. The vessel was cleared of thrombus and closed with continuous suturing. The skin was closed with single interrupted stitches. Over the following three days, the level of respiratory support could be reduced safely.

The patient was discharged from hospital after 12 weeks. Although he initially presented with incomplete tetraplegia, which was mainly symptomatic in his right arm, a generally improved condition allowed him to walk again and to use his left arm without limitations.

## Discussion

In several cases, we observed that lung injuries and excessive, prolonged ventilation caused ECMO to be initiated too close to the point at which ARDS became irreversible. We therefore support the early use of venovenous ECMO after major trauma. We usually run our treatment in major trauma patients with low dose heparin (400iu/h), especially in this case as the patient had a haematoma in the spinal canal after a spinal contusion with incomplete neurological paraplegia. In internal medicine settings, patients are generally treated with therapeutic doses of heparin to avoid thromboembolic events.^9^ Nevertheless, in a different setting such as in major trauma, it is possible to run the ECMO with low dose heparin or even without heparin, which does not cause a higher incidence of clot formation or thromboembolic events.^10^

An Avalon Elite™ cannula was used as we planned early mobilisation for the patient and as it avoids the risk of reperfusion. In addition, the insertion procedure was briefer than for other cannulas and injured only one vessel. Since only one DLC is used with the Avalon Elite™ procedure, it is critical to ensure that the cannula is positioned perfectly. Furthermore, it is necessary to confirm that the cannula is set to a rotation angle at which the infusion port points precisely towards the tricuspid valve. If the blood gas parameters deteriorate under ECMO therapy, one should first confirm the position of the cannula and perform any necessary corrections immediately.

It is highly possible that manipulations performed after an initial malpositioning of the cannula under low dose heparin will lead to adherence and thrombosis. This adherence caused by a thrombus cannot be revealed by CT as it occurs in a tiny margin between the outer surface of the cannula and the inner vascular surface.

## Conclusions

When using a single cannula such as the Avalon Elite™, the proper initial placement is critical for avoiding further complications following subsequent repositioning. The issue of effective anticoagulation in major trauma patients receiving ECMO support must be decided on an individual basis. As discussed above, any resistance that occurs during an attempt to remove a cannula can lead to serious complications. CT cannot illustrate this kind of thrombotic adherence. In this case involving the formation of local peripheral thrombi, a sternotomy could be avoided and an approach by peripheral skin incision was sufficient for the safe removal of the cannula.
